# Apoptosis, ageing and cancer susceptibility

**DOI:** 10.1038/sj.bjc.6600767

**Published:** 2003-02-18

**Authors:** R S Camplejohn, R Gilchrist, D Easton, E McKenzie-Edwards, D M Barnes, D M Eccles, A Ardern-Jones, S V Hodgson, P M Duddy, R A Eeles

**Affiliations:** 1 Richard Dimbleby Department of Cancer Research, Guy's, King's and St Thomas' School of Medicine, St Thomas' Hospital, London SE1 7EH, UK; 2 Cancer Research UK (formerly CRC) Genetic Epidemiology Unit, Strangeways Research Laboratory, Worts Causeway, Cambridge CB1 8RN, UK; 3 Cancer Research UK (formerly ICRF) Breast Pathology Laboratory, Guy's Hospital, London SE1 9RT, UK; 4 Wessex Clinical Genetics Service, Princess Anne Hospital, Southampton SO16 5YA, UK; 5 Institute of Cancer Research & Royal Marsden NHS Trust, Downs Road, Sutton, Surrey SM2 5PT, UK; 6 Clinical Genetics, Guy's Hospital, London SE1 9RT, UK

**Keywords:** apoptosis, radiation, cancer susceptibility, age

## Abstract

We have previously shown that peripheral blood lymphocytes (PBL) from individuals carrying a germline *TP53* mutation show a dramatically reduced apoptotic response to radiation. As part of a study of this phenomenon, we also investigated apoptotic response in a series of breast cancer patients lacking *TP53* mutations and in a control group of individuals without cancer. There was a significant reduction in mean apoptotic response with increasing age in all groups. These findings are consistent with a number of studies in rodents, which have demonstrated a reduction in DNA damage-induced apoptosis with increasing age. In addition, after adjusting for age, breast cancer patients showed significantly reduced apoptotic responses compared with normal controls (*P*=0.002). The odds ratio for breast cancer in women with an apoptotic response of <35%, compared with women with a response of >49%, was 6.42 (95% CI 1.68–24.6). The data further support the hypothesis that a reduction in apoptotic response to DNA damage with increasing age may play a significant role in the age-related increase in cancer.

A number of recent studies have shown a reduced apoptotic response to DNA damage with increasing age in rodents. For example, Polyak *et al* (1997) observed a reduced apoptotic response of lymphocytes to 5 Gy radiation in mice with increasing age. Suh *et al* (2002) found a reduced apoptotic response in rat liver cells exposed to methyl methane-sulphonate. Both of these papers discuss the possibility that the sharp rise in cancer incidence (and perhaps other diseases) with age may be partly related to a systemic failure of apoptosis.

We have previously published results concerning apoptotic response to gamma radiation in peripheral blood lymphocytes (PBL) from carriers of germline *TP53* mutations. Cells from such individuals have a defective apoptotic response when subjected to gamma radiation (Camplejohn *et al*, 1995,^2000^; Camplejohn *et al*, 2001). The reduction in apoptotic response is such that there is essentially no overlap in the response distributions in *TP53* mutation carriers and normal controls (12 *vs* 46% mean response, *P*<0.0001 in the two groups in Camplejohn *et al* (2000)). This reduction in apoptotic response exactly mirrors that seen in thymocytes from heterozygous *TP53* knockout mice (Clarke *et al*, 1993; Lowe *et al*, 1993). We now report results on almost 500 individuals including a group of female breast cancer patients lacking a *TP53* mutation, a group of unaffected individuals from cancer-prone families and a normal control group. These data have allowed us to investigate changes in apoptotic response with increasing age and to compare response in the group of breast cancer patients with controls.

## MATERIALS AND METHODS

### Subjects

For the purposes of the analyses reported in this paper, individuals with a germline *TP53* mutation were excluded. To achieve this, all individuals with a clinical history consistent with Li–Fraumeni syndrome/Li–Fraumeni-like syndrome (LFS/LFL), and individuals with a very low apoptotic response (<32%) were screened using sequencing and/or the FASAY, a functional assay of *TP53* status (Duddy *et al*, 2000). Samples were obtained from three main groups of individuals: (i) Normal controls (*n*=75), who were either patients or members of hospital staff, had never had cancer and whose families had no unusual history of the disease. (ii) Breast cancer patients who attended either the Genetics clinic at the Royal Marsden Hospital or the Breast Cancer Unit at Guy's Hospital. Owing to the referral patterns for these clinincs, this group was biased towards patients with a family history of breast cancer. Of the 243 female breast cancer patients, 72 germline *BRCA1* and five *BRCA2* mutations were detected. (iii) Members of cancer-prone families (*n*=80), who themselves had not had cancer. This group was heterogeneous, consisting of members of *BRCA1, BRCA2* and other cancer predisposing mutation families, some of whom carried the mutation in question and some of whom did not. In addition, included in this group were individuals from families with multiple cases of cancer for whom the genetic basis of this susceptibility was unknown. Finally, the apoptotic assay was performed on a small number of patients with cancers other than breast cancer (*n*=39) and on four male breast cancer patients (Table 2). All patient samples were taken with written informed consent in accord with local ethical committee approval.

### Apoptotic assay

#### Separation and culture of PBL

Whole blood (25 ml) was collected in preservative-free heparinised tubes and taken as rapidly as possible to the laboratory for separation of mononuclear cells. After removal of plasma, PBL were separated by centrifugation on Histopaque (Sigma, Dorset, England), washed and resuspended in 10 ml RPMI 1640 medium (Gibco, Paisley, Scotland) containing 10% serum plus antibiotics. Cell concentrations were determined using a Casy Counter (Schaerfe System, Reutlingen, Germany) and the concentration adjusted by addition of medium so as to achieve a concentration of 5×10^5^ PBL ml^−1^. A 10 ml measure of this suspension was added to a series of Falcon T25 tissue culture flasks (Becton Dickinson, Oxford, England) and the cells were cultured for 70 h and either irradiated or mock-treated. Following a further 24 h, culture cells were split into three aliquots and fixed in 70% ethanol.

#### Irradiation procedure

**Figure 1:**
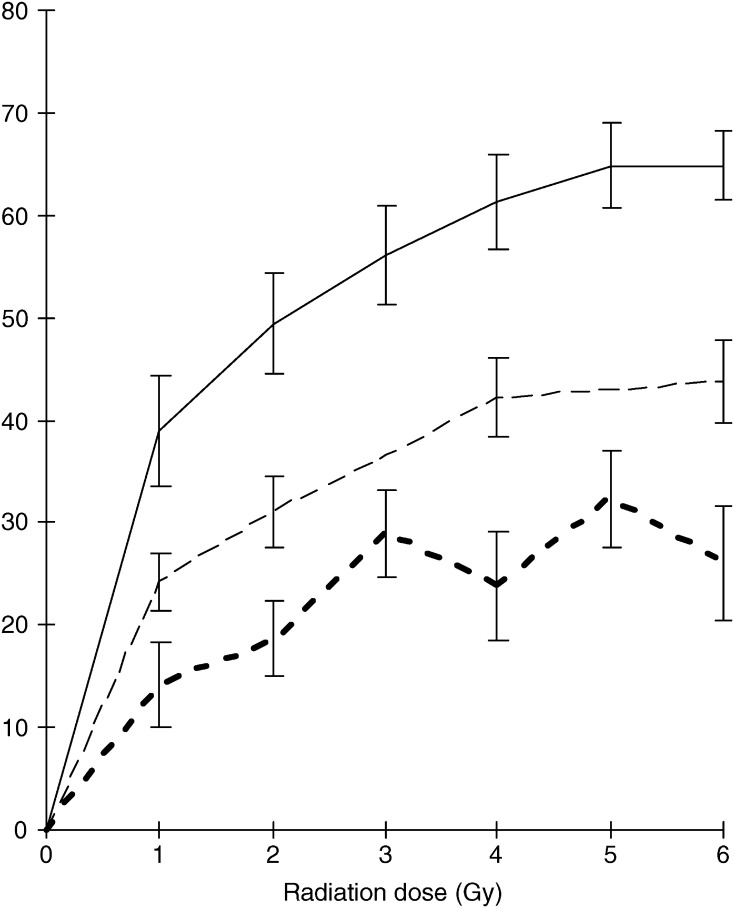
The above graph plots mean radiation-induced apoptosis as a percentage of total PBL in a control group (solid line, *n*=6), a group of general breast cancer patients (dashed line, *n*=5) and, for comparison, a group of LFS/LFL TP53 mutation carriers (thick dashed line, *n*=6). Error bars show the s.e. between each data measurement.

Irradiation was carried out using a Gammacell 1000 Elite (Nordion International Inc., Ontario, Canada) containing a caesium 137 source and with a dose rate of 858 cGy min^−1^. Cells were subjected to a dose of 4 Gy except in the dose–response experiment outlined below. A total of 4 Gy was chosen as a dose of radiation, which induces a maximum apoptotic response in all groups of individuals (Figure 1

). To produce the data in Figure 1, 50 ml of blood was obtained from a series of normal individuals, breast cancer patients lacking a germline *TP53* mutation and, for comparison, a group of *TP53* mutation carriers. PBL were separated and treated as described above except that aliquots of cells were exposed to radiation doses between 0 and 6 Gy.

#### Analytical flow cytometry

After removal of ethanol, 2×10^6^ cell aliquots were subjected to treatment with 0.1 M Hcl at 37°C for 12 min to extract low molecular weight DNA. After washes, cells were stained for DNA content by addition of propidium iodide (PI-Sigma) at a final concentration of 50 *μ*g ml^−1^ and RNAse (Sigma) at a final concentration of 250 *μ*g ml^−1^ in a volume of 1 ml. Cells were stained for a minimum of 30 min prior to measurement of red fluorescence (PI), forward and 90° light scatter on a FACSCalibur flow cytometer (Becton Dickinson, Oxford, England). At least 10 000 cells per sample were scanned and data stored in list mode prior to analysis using CellQuest software. Doublet discrimination using pulse area/width analysis on the PI signal was used to remove cell clumps from the analysis.

#### Measurement of apoptosis

Measurement of the extent of apoptosis was performed by assessment of cells appearing in a sub-G1 peak on DNA profiles. The apoptotic response to radiation was defined as the increase in apoptosis seen when comparing the irradiated with the unirradiated sample (% apoptosis after 4 Gy−% apoptosis after 0 Gy). This flow cytometric method has been validated in many publications and by comparison in our laboratory with a number of other techniques including electron microscopic counting of apoptotic cells and cell sorting of apoptotic cells (Camplejohn *et al*, 1995).

### Statistical analysis

The apoptotic response measure used was found to be roughly normally distributed, and analyses were therefore performed on apoptotic response untransformed. The effect of age was first examined by linear regression. The effect of cancer status on apoptotic response was assessed by multiple regression. To adjust for age as a potential confounder, we included age as a covariate as <30, 30–39, 40–49, 50–59 or 60+ years. We estimated the odds ratios for cancer risk at different levels of apoptotic response using logistic regression. Apoptotic response was categorised as <35%, 35–40%, 40–49% and >49%, which defined four roughly equally frequent categories in the data set. Age (using the same five categories defined above) was again included as a covariate. Repeated measurements on the same individual were used to estimate the proportion of the variance because of individual variation, using analysis of variance. Analyses were performed using S-plus.

## RESULTS

The apoptotic response of PBL to a range of doses of ionising radiation is shown in Figure 1, for a subset of breast cancer patients, controls and, for comparison, a group of *TP53* mutation carriers. This small dose–response experiment demonstrates that cells from all three groups have a maximal apoptotic response at around 4 Gy.

There was a significant reduction in apoptotic response to 4 Gy irradiation in all groups of individuals with increasing age (Table 1

**Table 1:** Summary of the effect of age on apoptotic response to 4 Gy irradiation

	**Category of individual (mean % apoptotic response (number of individuals))**		
**Age (years)**	**Breast carcinoma**	**Unaffected with family history of cancer**	**Controls**
<30	48.9 (6)	47.9 (7)	52.4 (19)
30–39	42.2 (61)	44.7 (18)	48.2 (20)
40–49	37.8 (69)	40.6 (18)	43.7 (3)
50–59	37.6 (76)	36.1 (14)	44.3 (5)
⩾60	37.1 (31)	27.9 (7)	43.7 (1)

). Among the breast cancer patients, there was a marked decline with age (*P* trend=0.002), with the mean apoptotic response declining from 48.9% for cases aged below 30 years to 37.1% for cases aged 60 and over. A decline with age was also seen for unaffected individuals with a family history (*P*=0.0001), and a similar pattern was observed for the control group (*P*=0.08), although in this case the effect was less definite because of the small number of controls over the age of 40. Age-adjusted apoptotic response for male controls was lower than that for females (mean difference 7.06, *P*=0.01).

The mean apoptotic response at 4 Gy for each category is shown in Table 2

**Table 2:** Summary of apoptotic assay results for groups defined by gender and disease status

**Category of individual**	**Gender (number of individuals)**	**Mean apoptotic response to 4 Gy irradiation (% ±s.d.)**	Significance of difference with controls*
Control	Female (48)	49.1±10.2	—
	Male (27)	43.1±12.0	—
Breast carcinoma	Female (243)	39.0±11.0	*P*=0.002
	BRCA1/2+ve (77)	37.4±11.3	
	BRCA1/2−ve (166)	39.8±10.8	
	Male (4)	33.2±5.16	NA
Sarcoma	Female (8)	37.3±17.0	*P*=0.008
	Male (15)	49.3±8.5	NS
Other carcinoma	Female (11)	32.7±9.5	*P*=0.007
	Male (5)	44.7±10.9	NS

* Significance of difference in mean response compared with controls, after adjusting for age.

. After adjusting for age, breast cancer cases had a significantly lower mean apoptotic response than unselected normal controls (adjusted mean difference 5.74, *P*=0.005). Among the breast cancer cases, the age-adjusted mean score was slightly lower for *BRCA1/2* carriers than noncarriers (mean difference 3.77, *P*=0.014). After excluding known *BRCA1/2* carriers, the difference in mean score between cases and controls was reduced (mean difference 3.61, *P*=0.11). Interestingly, the mean apoptotic response for male breast cancer patients was lower than that for female breast cancer patients, but this result was based on only four patients.

The estimated odds ratios for breast cancer by level of apoptotic response, after adjusting for age by logistic regression, are shown in Table 3

**Table 3:** Risks by category of apoptotic response

**Category of apoptotic response to 4 Gy irradiation (%)**	**Breast cancer cases (risk (95% confidence interval) (number in each group)**
>49	1.0 (*n*=46)
40–49	1.20 (0.48–3.00) (*n*=53)
35–40	1.67 (0.56–5.02) (*n*=57)
<35	6.42 (1.68–24.6) (*n*=87)

. The most marked effect is for apoptotic response <35%, which is associated with an estimated odds ratio of 6.42 (95% CI 1.68–24.6) compared with individuals with a score of >49% (*P* trend=0.005).

## DISCUSSION

Mean apoptotic response reduced with increasing age in all groups (Table 1). This was seen clearly in the breast cancer cases and in unaffected women with a family history of cancer, but was also apparent in the unrelated control group, although the number of older controls was small. Polyak *et al* (1997) observed a reduced apoptotic response of lymphocytes to 5 Gy radiation in mice as they aged, and suggested that similar data on humans would be of interest. Suh *et al* (2002) found a reduced apoptotic response in rat liver cells exposed to methyl methane-sulphonate. Both of these papers discuss the possibility that the sharp rise in cancer incidence (and perhaps other diseases) with age may be partly related to a systemic failure of apoptosis. The age distribution of breast cancer incidence in humans is usually attributed to the fact that several rate-limiting somatic mutational events are required to generate a malignant tumour (Armitage *et al*, 1961), with a slowing down of the carcinogenic process at the menopause because of the lower mitotic rate in postmenopausal women. However, in a recent study (Peto *et al*, 2000), an alternative model was proposed in which a woman's breast cancer incidence rises sharply to a high constant rate at a genetically determined age. Systemic failure of DNA repair processes, including apoptosis, might be one of the mechanisms behind such a model.

This possibility is supported by the finding that the group of 243 female breast cancer patients without a known *TP53* mutation showed an unexpected and statistically significant (*P*=0.002) reduction in apoptotic response to DNA damage compared to controls. Many of the genes involved in the apoptotic pathway have been documented, and polymorphisms in these or related genes may explain the observed variability in apoptotic response. Perhaps reflecting this, there is some evidence of *BRCA1* and *BRCA2* mutation carriers having a lower apoptotic response than other breast cancer patients. Alternatively, it is possible that the decreased apoptotic response may be caused by the onset of cancer, or the treatment of it, rather than preceding it. Prior therapy is a potential problem with this study in that 134 of the 243 female breast cancer patients had radiotherapy or some type of systemic therapy at various times prior to the apoptotic assay being performed. However, apoptotic response for the group of patients receiving no such therapy (mean apoptotic response=40±11% s.d.) was virtually identical to that for the whole group (39.0±11.0% s.d.) and thus it does not seem likely that therapy had a significant effect on apoptotic response. The general idea that apoptotic response to DNA damage and susceptibility to cancer are linked is supported by work in mice. Splenocytes from C57BL/6 mice show a large and rapid apoptotic response to 1 Gy radiation, in comparison with splenocytes from DBA/2 mice, in which the response is slower and reduced in extent (Wallace *et al*, 2001). Radiation-induced malignancy is greater in DBA/2 as compared with C57BL/6 mice. Nevertheless, with regard to the link between apoptotic response and breast cancer in humans, a larger case–control study based on incidence cases without systemic treatment and with better age matching of controls would be needed to resolve any uncertainty.

The finding of a statistically significant reduction in apoptotic response with age in humans supports the hypothesis set out by Polyak *et al* (1997) and Suh *et al* (2002), based on rodent data, that such a reduction may play an important role in age-associated disease processes. In relation to cancer specifically, this hypothesis is further supported by the reduced apoptotic response seen in breast cancer patients *vs* normal controls.
